# Supplementary material for: Methylglyoxal Impairs Sister Chromatid Separation in Lymphocytes
Source: Int J Mol Sci. 2022 Apr 8;23(8):4139. doi: 10.3390/ijms23084139 (PMC9030103; doi:10.3390/ijms23084139)
Supplement: Supplementary file 1 [file ijms-23-04139-s001.zip › Supplementary 2.pdf]

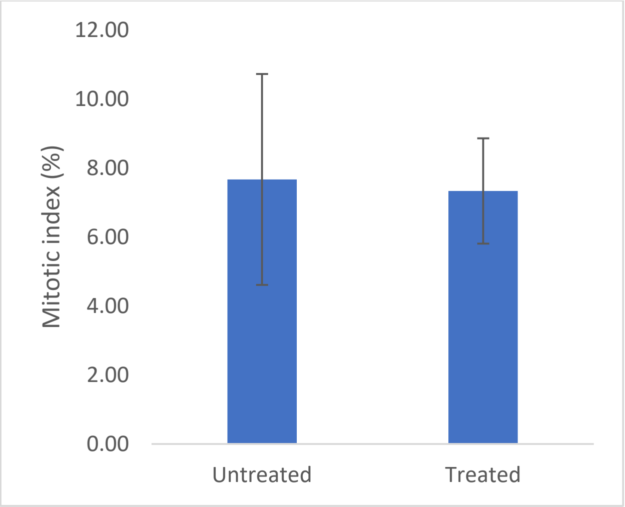

Supplementary Figure S1. Methylglyoxal does not affect the mitotic index of WIL2-NS cells. Data are mean ± SD of *n*=3.
